# The changing landscape of phase II/III metastatic sarcoma clinical trials—analysis of ClinicalTrials.gov

**DOI:** 10.1186/s12885-018-5163-2

**Published:** 2018-12-13

**Authors:** Y Que, W Xiao, BS Xu, XZ Wen, DS Weng, X Zhang

**Affiliations:** Department of Medical Melanoma and Sarcoma, State Key Laboratory of Oncology in South China, Collaborative Innovation Center for Cancer Medicine, Sun Yat-sen University Cancer Center, 651 East Dongfeng Road, Guangzhou, 510060 China

**Keywords:** Sarcoma, ClinicalTrials.gov, Target therapy, Immunotherapy, Landscape

## Abstract

**Background:**

Well-designed clinical trials are of great importance in validating novel treatments and ensuring an evidence-based approach for sarcoma. This study aimed to provide a comprehensive landscape of the characteristics of metastatic or advanced sarcoma clinical trials using the substantial resource of the ClincialTrials.gov database.

**Methods:**

We identified 260,755 trials registered with ClinicalTrials.gov in the last 20 years, and 277 of them were eligible for inclusion. The baseline characteristics were ascertained for each trial. The trials were systematically reviewed to validate their classification into 96 trials registered before 2008 and 181 trials registered between 2008 and 2017.

**Results:**

We found that in the last decade, metastatic and advanced sarcoma trials were predominantly phase II-III studies (*p* = 0.048), were more likely to be ≥2 arms (17.7% vs 35.3%, respectively; *p* = 0.007), and were more likely to use randomized (13.5% vs 30.4%; *p* = 0.002) and double-blinded (2.1% vs 9.4%; *p* = 0.024) assignment than trials registered before 2008. Furthermore, in the last 10-year period, metastatic sarcoma trials were more likely to be conducted in Asia. Treatment involving target therapy and immunotherapy were more common (71.8% vs 37.5%; *p* < 0.001) than in previous years.

**Conclusions:**

Our data showed provocative changes in the sarcoma landscape and demonstrated that the incidence of clinical trials with target therapy and immunotherapy is increasing. These findings emphasize the desperate need for novel strategies, including target therapy and immunotherapy, to improve the outcomes for patients with advanced sarcoma.

**Electronic supplementary material:**

The online version of this article (10.1186/s12885-018-5163-2) contains supplementary material, which is available to authorized users.

## Background

Bone and soft-tissue sarcoma is a heterogeneous group of many rare tumours that comprises more than 50 subtypes [1, 2]. The incidence of soft-tissue sarcomas in the US is approximately 11,280 cases every year, and in Europe, the estimated incidence rate of sarcomas taken as a whole is 5 cases per 100,000 people per year [3]. Doxorubicin and ifosfamide remain the most effective chemotherapy drugs available for the first-line treatment of advanced or metastatic sarcoma patients [4, 5]. However, the prognosis and the long-term outcome remain unsatisfactory. Therefore, improvements in patient outcome and the development of new therapies for bone and soft-tissue sarcomas through clinical trials are urgently needed. In the last decade, we have witnessed the rapid development and success of precision medicine based on individual characteristics in sarcoma. Efforts have been made to develop targeted therapeutic agents that reflect specific molecular biology, from pazopanib, which has significantly increased progression-free survival by a median of 3 months compared with placebo [6], to a great improvement in overall survival and acceptable toxicity in patients treated with olaratumab with doxorubicin. Additionally, immunotherapeutic approaches in sarcoma hold substantial potential, from Coley’s toxin to adoptive cell therapy [7, 8]. Thus, the advances of promising approaches have ushered in an era of innovative therapies. In the present study, we aimed to explore the landscape of clinical trials of sarcoma over time to better understand what changes in clinical trial design have been made and the trends of new therapies for sarcoma over the last 2 decades.

We reviewed all the metastatic phase II/III bone and soft-tissue sarcoma clinical trials present on ClinicalTrials.gov. The ClinicalTrials.gov website is the largest clinical trial registry, with over 200,000 trials, and is a publicly available database that was developed and is maintained by the National Library of Medicine [9]. In 2007, Congress expanded ClinicalTrials.gov by requiring additional trial information [10]. Because phase I trials have focused on the safety of systemic treatments and not as much on potential innovations in the medical treatment process, we restricted the present analysis to phase II and III clinical trials for sarcoma registered on ClinicalTrials.gov. We sought to describe the emerging landscape and characteristics of such metastatic or locally advanced sarcoma phase II and III trials conducted within the last 2 decades.

## Methods

### Creation of the ClinicalTrials.gov dataset

**Fig. 1:**
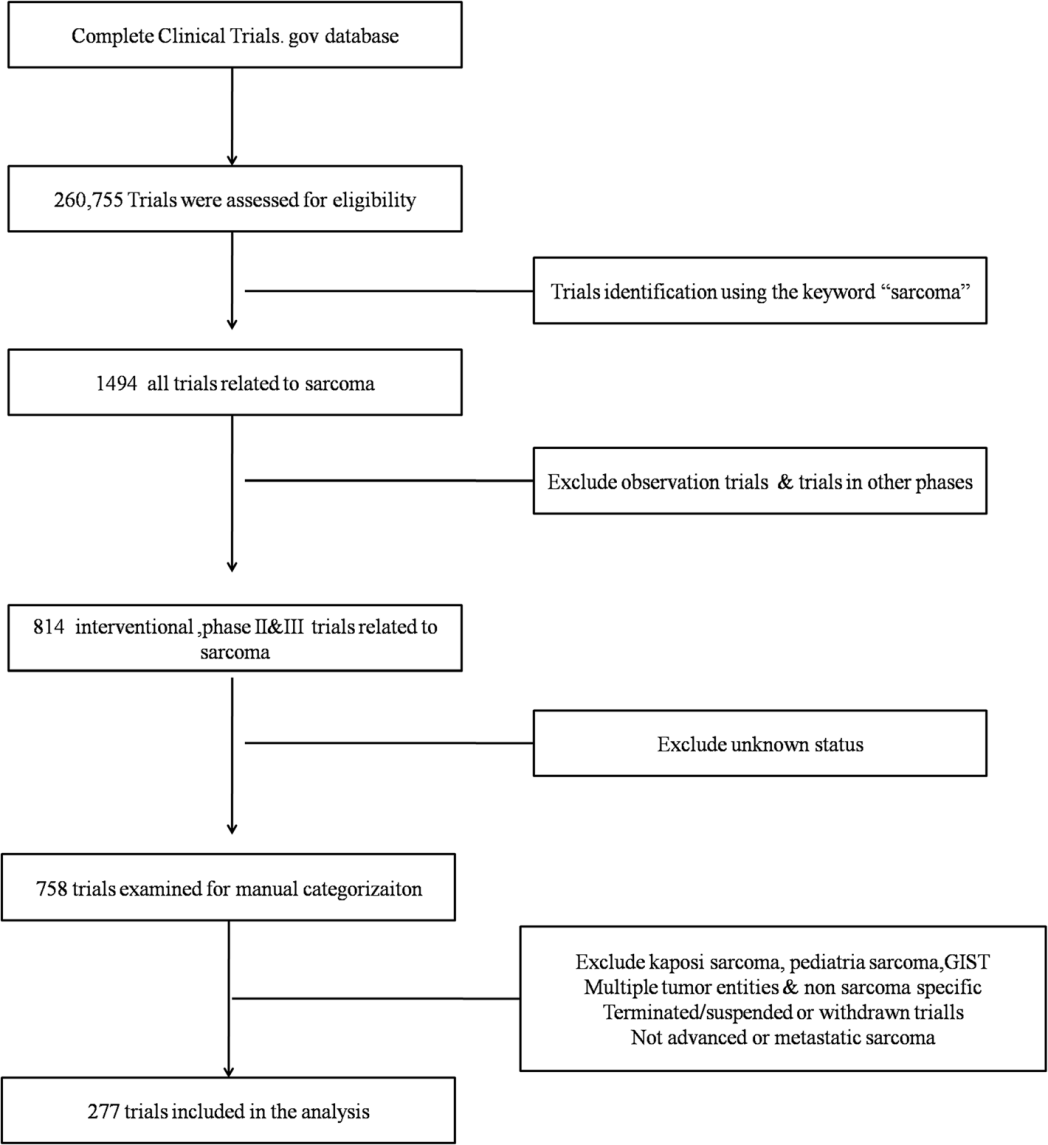
Flow chart for selection of trials included in the study. GIST, Gastrointestinal stromal tumours

On December 7, 2017, a dataset of 260,755 studies registered on ClinicalTrials.gov was downloaded. We queried ClinicalTrials.gov for the term “sarcoma” in the short titles, scientific titles, conditions, short summaries and detailed descriptions. Using this search strategy, 1494 trials were identified. We restricted our search to interventional trials and trials in phases II and III with a “known” status. We excluded trials with multiple tumour entities or that were non-sarcoma specific as well as those in a terminated/suspended or withdrawn status. Kaposi’s sarcoma, paediatric sarcoma, gastrointestinal stromal tumor (GIST) and sarcomas that were not advanced or metastatic were also excluded. After examination for manual categorization, 277 trials were selected for analysis. The trial selection process is shown in Fig. 1.

The extracted data elements included the following: phase (II-III), sponsor, study site locations, conditions (disease type), interventions, enrolment, study design, trial start date, and number of participating centres. The sponsor type (NIH, industry or other) for each trial was determined using a previously published algorithm [11].

All the registered trials were classified according to their specific treatment and were divided into four groups: those using a targeted drug, those using immunotherapy, those using chemotherapy and others. The treatments were categorized based on the resources available in the following databases: www.drugbank.com [12], pubchem.ncbi.clm.nih.gov, the National Cancer Institute Dictionary of Cancer Terms (www.cancer.gov), the Scopus database, the PubMed Database, and Google Scholar. Because of data unavailability, we manually classified the drugs according to the WHO guidelines. In addition, the specific target receptors were also investigated and noted.

### Statistical analysis

A descriptive analysis was performed to provide an overview of the emerging landscape of metastatic and advanced phase II and III sarcoma clinical trials registered on ClinicalTrials.Gov. The characteristics of the trials conducted between 2008 and 2017 were compared with those conducted before 2007 using Chi-squared or Fisher’s exact tests. All the data were analysed using SPSS 18.0. All *p-*values were 2-tailed with significance defined as *p* < 0.05.

## Results

### Clinical trial characteristics

Of the initial 260,755 trials identified, 277 were eligible for inclusion in the analysis. The reasons for exclusion are shown in Fig. 1. Trial names with ClinicalTrials.gov identification numbers are listed in the supplemental data for eligible trials in this study (Additional file 1). The trial design characteristics are presented in Table 1. Of the metastatic or advanced sarcoma trials with a sponsor, 11.9% were sponsored by NIH, 21.7% were sponsored by industry and 66.4% were sponsored by others, including academic groups. The most common study region explored in the eligible trials was the US (67.1%), followed by Europe (23.5%) and Asia (9.4%).

**Table 1:** Trials characteristics

	Number	Percent
Trial phase		
Phase I/II	48	17.3
Phase II	200	72.2
Phase II/III	4	1.4
Phase III	25	9.0
Sponsor		
NIH	33	11.9
Industry	60	21.7
Other	184	66.4
Enrollment size		
0–50	136	49.1
51–100	65	23.5
101–200	40	14.4
201–300	6	2.2
301-more	16	5.8
NR	14	5.1
Number of centers		
1	97	35.0
2	6	2.2
multicenter	174	62.8
Number of arms		
1	196	70.8
2	67	24.2
≥3	14	5.1
Treatment allocation		
Non-randomized	209	75.5
Randomized	68	24.5
Masking		
Open-label	257	6.9
Single-blind	1	0.4
Double-blind	19	92.8
Region		
United states	186	67.1
Europe	65	23.5
Asia	26	9.4

Trial characteristics are based on the clinical trials which are advanced or metastatic sarcoma specific. Abbreviations: *NIH* National Institutes of Health, *NR* null value

**Fig. 2:**
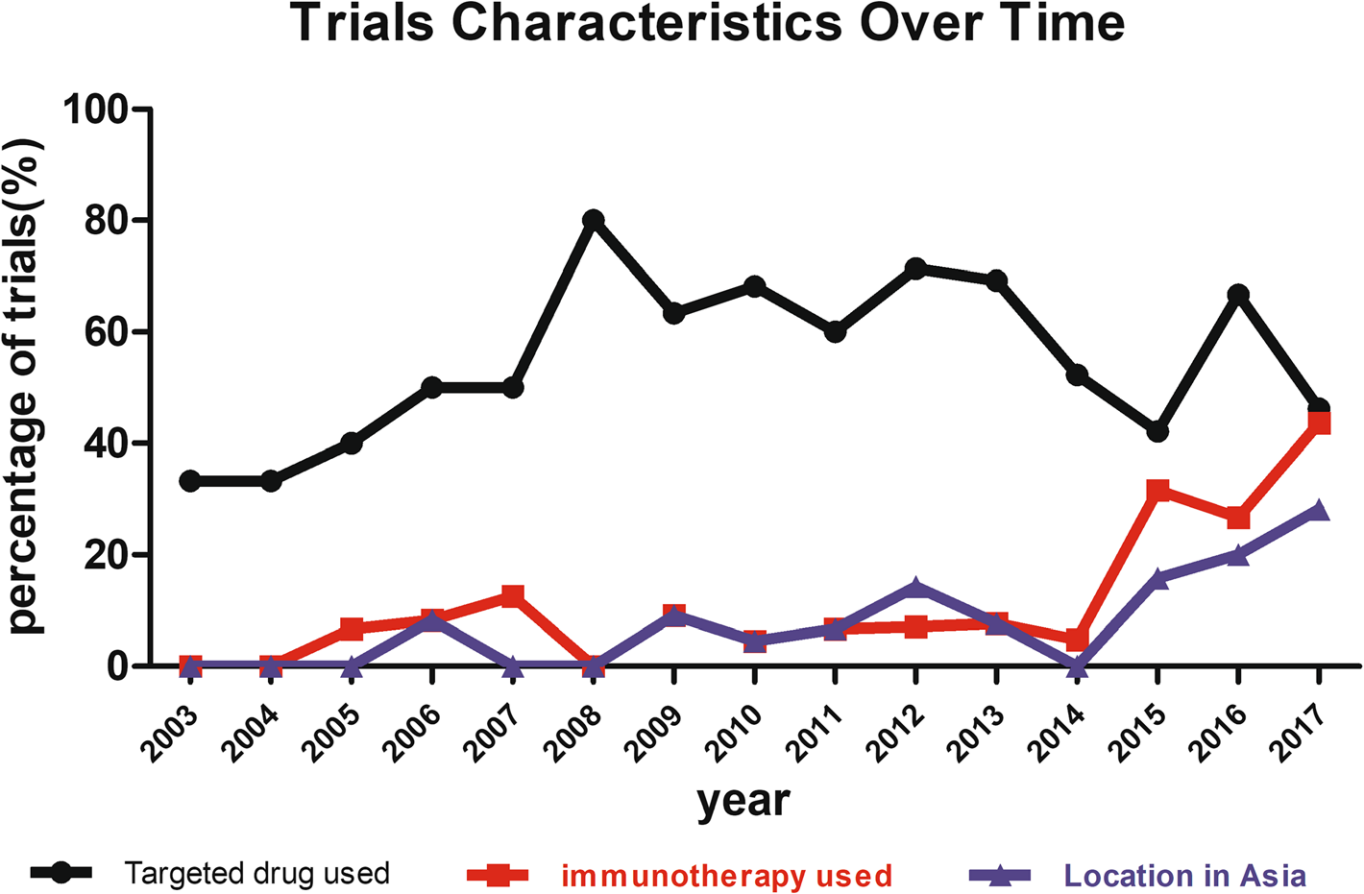
Trial characteristics of phase II-III clinical trials of metastatic sarcoma presented on ClinicalTrials.gov over time. The percentage of trials each year is shown. The number of target therapies has increased since 2003 and reached its peak in 2008. Between 2003 and 2014, the number of immunotherapy trials and trials initiated in Asia was small, while these proportions increased by 2014

The characteristics of the trials conducted in the last decade versus those conducted before 2008 are shown in Table 2. Compared with trials registered before 2008, those registered in the last 10 years were significantly more likely to be phase III (*p* = 0.048). In addition, in the last decade, sarcoma studies were more likely to contain more than 2 arms (17.7% vs 35.3%; *p* = 0.007) and to be randomized (13.5% vs 30.4%; *p* = 0.002) and double-blinded (2.1% vs 9.4%; *p* = 0.024). We also sought to identify trends in clinical trial treatments over this period. In the past 10 years, metastatic or advanced sarcoma clinical trials were more likely to involve target therapy and immunotherapy (71.8% vs 37.5%; *p* < 0.001) (Table 2). The percentage of trials involving immunotherapy increased from 12.5% in 2007 to 43.6% in 2017. The proportion of adult clinical sarcoma trials initiated in Asia increased from 0% in 2007 to 28.2% in 2017 (Fig. 2).

**Table 2:** Baseline characteristics of clinical trials associated with the changing year (*n* = 277)

Characteristics	Number of trials (%)		*P* value
	Before to 2007 year	2008–2017 year	
Trial phase			0.048
Phase I/II	10 (10.4)	38 (21.0)	
Phase II	78 (81.3)	122 (67.4)	
Phase II/III	0 (0)	4 (2.2)	
Phase III	8 (8.3)	17 (9.4)	
Sponsor			0.215
NIH	16 (16.7)	17 (9.4)	
Industry	20 (20.8)	40 (22.1)	
Other	60 (62.5)	124 (68.5)	
Number of centers			0.560
1	32 (33.3)	65 (35.9)	
2	1 (1.0)	5 (2.8)	
multicenter	63 (65.6)	111 (61.3)	
Number of arms			0.007
1	79 (82.3)	117 (64.6)	
2	13 (13.5)	54 (29.8)	
≥3	4 (4.2)	10 (5.5)	
Treatment allocation			0.002
Non-randomized	83 (86.5)	126 (69.6)	
Randomized	13 (13.5)	55 (30.4)	
Masking			0.024
Open-label	94 (97.9)	164 (90.6)	
Double-blind	2 (2.1)	17 (9.4)	
Region			< 0.001
United states	77 (80.2)	109 (60.2)	
Europe	18 (18.8)	47 (26.0)	
Asia	1 (1.0)	25 (13.8)	
Therapy			
Chemotherapy	55 (57.3)	40 (22.1)	< 0.001
Target therapy /immunology therapy	36 (37.5)	130 (71.8)	
other	5 (5.2)	11 (6.1)	

Percentage of trial characteristics with each inclusion criteria group. Chi-squared test was used for class variables NIH, National Institutes of Health

### Multi-RTK and VEGFR are prime targets in phase II/III clinical trials of advanced soft tissue sarcoma

Among the 277 eligible clinical trials, 128 trials were identified to involve target therapy among the metastatic or advanced sarcoma trials. An overview of all the targeted therapy receptors is presented in Table 3.

**Table 3:** Target therapy clinical trials related to phase II & III advanced or metastatic sarcoma

Targets	Trials (n)	%
Multi-RTK	40	31.3
VEGFR	17	13.3
mTOR	12	9.4
HDAC	9	7.0
Combination targets	8	6.3
CDK	6	4.7
IGF-1R	5	3.9
PDGFR	5	3.9
Hedgehog	3	2.3
AKT	2	1.6
CD105	2	1.6
EGFR	2	1.6
HER2	2	1.6
Hypoxic region	2	1.6
MET	2	1.6
26S proteasome	1	<1
Aurora A	1	<1
CRM1	1	<1
DR5	1	<1
Endoglin	1	<1
Endosialin/TEM1	1	<1
G6PD	1	<1
PPAR-γ	1	<1
SRC	1	<1
Tie2	1	<1
β-receptor	1	<1

The different targets of 128 registered clinical trials. RTK, receptor tyrosine kinase; VEGFR, vascular endothelial growth factor receptor; HDAC, histone deacetylase; CDK, cyclin-dependent kinase; IGF-1R, insulin-like growth factor 1 receptor; PDGFR, platelet-derived growth factor receptor; AKT, protein kinase B; EGFR, epidermal growth factor receptor; HER2, human epidermal growth factor receptor 2; MET, a receptor tyrosine kinase that is produced as a single-chain precursor; CRM1, chromosomal maintenance 1; DR5, death receptor-5; G6PD, glucose-6-phosphate dehydrogenase; PPAR-γ, peroxisome proliferator-activated receptors; SRC, proto-oncogene tyrosine-protein kinase; Tie2, tyrosine kinase with immunoglobulin-like and EGF-like domains 1

In the present study, we found that a growing number of multiple-RTK inhibitors are being tested for sarcoma treatment (40, 31.3%). This type of target agent included imatinib, sunitinib, sorafenib, pazopanib, regorafenib, anlotinib, etc. Particularly, pazopanib acts as a novel TKI and plays a key role in a broad spectrum of tumour types (13/40, 32.5%) during these trials. We also found that some clinical trials focused on the target VEGFR, which plays a central role in tumour angiogenesis and metastasis (17, 13.3%). The other studied targeted receptors or pathways were mTOR (12, 9.4%), HDAC (9, 7.0%), CDK (4, 7%) IGF-1R (5, 3.9%) and PDGFR (5, 3.9%).

Furthermore, the proportion of clinical trials focused on target therapy that were initiated in Asia increased in the past 10 years compared with clinical trials initiated before 2008 (0 vs 12). In particular, anlotinib (NCT02449343, NCT01878448) and apatinib (NCT0312846, NC03064243, NCT03104335, NCT02711007, NCT03163381) are two typical representatives of multi-RTK inhibitors and VEGFR inhibitors that were developed in China.

The trials involving two target drugs are presented in Table 4. These trials were all conducted in the past 10 years, with seven conducted in the US and one conducted in Europe.

**Table 4:** Combination of two targeted drug Trials

NCT No.	Drug1	Drug2	Targets	Year	Phase	Region	Results
NCT03114527	Ribociclib	Everolimus	CDK, mTOR	2017	Phase II	US	No
NCT02343172	HDM201	LEE011	MDM2, CDK	2015	Phase I| Phase II	US	No
NCT02008877	ganetespib	Sirolimus	HSP90, CD105	2013	Phase I|Phase II	US	No
NCT01804374	Sorafenib	Everolimus	Multi-RTK, mTOR	2011	Phase II	Europe	No
NCT01281865	everolimus	imatinib	Multi-RTK, mTOR	2011	Phase I|Phase II	US	Yes
NCT01206140	Selumetinib	Temsirolimus	MEK, mTOR	2010	Phase II	US	Yes
NCT01016015	Cixutumumab	Temsirolimus	mTOR, IGF-1R	2009	Phase II	US	Yes
NCT00937495	vorinostat	bortezomib	HDAC,26S proteasome	2009	Phase II	US	Yes

Abbreviations: *CDK* cyclin-dependent kinase, mTOR mammalian target of rapamycin, *MDM2* Mouse double minute 2 homolog, *HSP90* heat shock protein 90, *RTK* receptor tyrosine kinase, *MEK* Mitogen-activated protein kinase kinase, *IGF-1R* Insulin-like growth factor 1 receptor, *HDAC* Histone deacetylase

### Immune checkpoint inhibition therapies contributed greatly in all clinical trials that evaluated immunotherapy

Thirty-three trials evaluated immunotherapy in metastatic or advanced sarcoma. Seventeen trials explored the role of immune checkpoint receptors (51.5%). Among them, 11 trials focused on the PD1 antibody (NCT0321745, NCT0312376, NCT3013127, NCT03056001, NCT3138061, NCT3069378, NCT02888665, NCT02691026, NCT02406781, NCT2301039, NCT03282344). Four trials evaluated anti-PD-L1 therapy (NCT03141684, NCT03074318, NCT03233698, NCT02609984), and two trials used the PD1 antibody combined with the CTLA-4 antibody as treatment (NCT02982486, NCT02428192). Seven (21.2%) trials evaluated vaccines as interventions in sarcoma. Five (15.2%) trials evaluated adoptive cell transfer as a treatment in advanced sarcoma patients. Only two (6.1%) of the eligible trials explored immunomodulators and oncolytic viruses (Table 5). The number of clinical trials focused on immunotherapy is not large compared with the number of clinical trials focused on target therapies, but the number of trials focusing on immunotherapy grew significantly since 2014 (Fig. 2). These results indicated that immunotherapy, especially the anti-PD1/PDL1 pathway, could be a trend in STS treatment. in STS.

**Table 5:** Immunotherapy Trials

Immunotherapy	Trials (n)	%
Immune checkpoint inhibitors	17	51.5
Vaccine	7	21.2
Adoptive cell transfer	5	15.2
Immunomodulator^a^	2	6.1
Oncolytic virus	2	6.1

^a^Immunomodulators are the active agents of immunotherapy. They are a diverse array of recombinant, synthetic, and natural preparations including interleukins, cytokines, chemokines

### The combination of immunotherapy and target therapy has increased in recent years

Because target therapy and immunotherapy are trends in sarcoma research, we evaluated the treatment combining immunotherapy with target therapy in advanced sarcoma patients. All five trials evaluated the PD1 antibody combined with targeted drugs, and one trial evaluated nivolumab combined with pazopanib in soft-tissue sarcoma (NCT03149120); one trial evaluated nivolumab combined with the mTOR inhibitor rapamycin in undifferentiated pleomorphic sarcoma, liposarcoma, chondrosarcoma, osteosarcoma and Ewing’s sarcoma (NCT03190174); one trial explored the role of the combination of pembrolizumab with axitinib in mostly alveolar soft-tissue sarcoma (NCT02636725); one trial evaluated nivolumab combined with sunitinib in soft-tissue sarcoma (NCT03277924); and the last trial, which is an open-label, phase II trial located in China, studied anti-PD1 therapy (camrelizumab) plus apatinib mesylate in locally advanced, unresectable or metastatic osteosarcoma (Table 6).

**Table 6:** Combination of target therapy and immunotherapy

NCT No.	Drug 1	Drug2	Conditions	Phase	region	Start year	results
NCT03149120	Nivolumab	Pazopanib	Soft Tissue Sarcomas	Phase II	US	2017	No
NCT03190174	Nivolumab	Rapamycin	Undifferentiated Pleomorphic Sarcoma|Liposarcoma|Chondrosarcoma|Osteosarcoma|Ewing Sarcoma	Phase I| Phase II	US	2017	No
NCT02636725	Pembrolizumab	Axitinib	Alveolar Soft Part Sarcoma|Soft Tissue Sarcomas	Phase II	US	2016	No
NCT03359018	Camrelizumab	Apatinib	osteosarcoma	Phase II	Asia	2018	No
NCT03277924	Nivolumab	Sunitinib	Soft Tissue Sarcoma|Bone Sarcoma	Phase I| Phase II	Europe	2017	No

Note: Nivolumab and pembrolizumab are PD-1 antibodies while camrelizumab is PD-L1 antibody

## Discussion

The development of new systemic treatments for soft-tissue sarcomas has progressed little in the past few decades, except for treatments for gastrointestinal stromal tumours. Patients with metastatic soft-tissue sarcomas have a median overall survival of approximately 12 months [6, 13]. Primarily, chemotherapy with doxorubicin and/or ifosfamide is a common approach, but some subtypes are resistant to chemotherapy [14]. The combination of gemcitabine and docetaxel is mainly used in leiomyosarcomas, and some retrospective analyses have confirmed the activity of this regimen in undifferentiated pleomorphic sarcomas of metastatic patients [15, 16]. In addition, trabectedin has been demonstrated to achieve a dimensional response or disease stability in more than 40% of patients, as evidenced by the longer OS in so-called L-sarcoma (liposarcoma and leiomyosarcoma) [17]. From these results, different cytotoxic drugs have shown specific activity in selective histotypes. However, many patients ultimately die because of limited alternate chemotherapeutic regimens for recurrent and metastatic diseases [18]. Thus, novel agents with new therapies and rigorous prospective clinical trials are desperately needed to validate the role of innovative strategies.

Our study demonstrated that sarcoma trials in the last 10 years were predominantly late-phase studies with a generally high proportion of multi-arm, randomized and double-blind studies. The most likely cause for this trend is the development of new monitoring processes to improve randomized trial performance, data collection and good clinical practice (GCP) compliance, such as PRIME—Peer Review Intervention for Monitoring and Evaluating sites [19]. Our analysis confirms the critical role for science in the establishment and performance of clinical trials. These findings likely reflect the trend and necessity for developing more randomized and double-blinded studies under GCP management.

We also found the growing role of target therapy and immunotherapy in bone and soft-tissue sarcoma trials by evaluating a comprehensive landscape. This may be the result of an increasing number of RTKs and other small molecular inhibitors that have been developed, and evidence is emerging that targeting RTKs and other small molecular inhibitors could be an effective approach. In the past few years, research on sarcoma has mostly focused on the molecular aspects of tumourigenesis [20, 21]. Imatinib was the first target TKI developed and approved by the Food and Drugs Administration (FDA) and European Medicines Agency (EMA). Adjuvant imatinib significantly improved recurrence-free survival compared with placebo (98% [95%CI 96–100] vs 83% [78–88] at 1 year after the resection of primary GIST [22]. After the production of imatinib, other promising TKIs have been utilized in clinical trials and released to the market, including sunitinib (NCT00400569, NCT00474994), sorafenib (NCT00238121, NCT00245102, NCT00217620) and pazopanib (NCT00753688). Pazopanib showed promising potential in the treatment of patients with STS. In the randomized, double-blind clinical trial (PALETTE-EORTC 62072), patients with metastatic STS of various histologies were randomly assigned to receive pazopanib or placebo. The results showed that the median progression-free survival was 4.6 months for the pazopanib group compared with 1.6 months for the placebo group, which indicated that pazopanib is a promising treatment option for patients with STS [6]. Thus, the US FDA and EMA approved pazopanib for use in the second or further line of treatment of patients with nonadipogenic STS [23]. Moreover, sarcoma trials are a new trend in Asia. Anlotinib is a novel multi-TKI that has been initiated in clinical trials in China and has shown some antitumour activity in several STS entities, including leiomyosarcoma (LMS), synovial sarcoma (SS) and alveolar soft tissue sarcoma (ASPS) [24]. Recently, a growing number of phase II/III clinical trials of apatinib, which is a new type of VEGFR inhibitor for sarcomas, have also been initiated in China (NCT0312846, NC03064243, NCT03104335, NCT02711007, NCT03163381). In addition, clinical trials focusing on other promising VEGFR inhibitors, such as cabozantinib (NCT01755195, NCT01979393), and tivozanib (NCT01782313), are ongoing and recruiting patients. It is notable that doxorubicin combined with olaratumab, the monoclonal antibody that specifically binds PDGFR, showed promising improvement in overall survival in patients with advanced STS compared with doxorubicin alone (NCT01185964). The rising trend of target therapy in sarcoma indicates a step forward in the concept of new developments in molecular targeted agents that significantly change the medical approach towards STS.

Additionally, a growing number of clinical trials investigating immunotherapy, particularly immune checkpoint inhibitors, are currently ongoing thanks to the success of anti-PD1 therapy in other tumours, including melanoma and NSCLC. Sarcomas are among the first tumours to be considered for immunotherapy [25]. From Coley’s toxin to adoptive cell therapy to immune checkpoint inhibitors, immunologic treatments hold substantial potential in sarcoma. In a randomized, double-blind phase study (SARC028), pembrolizumab suggested a potential benefit for undifferentiated pleomorphic sarcoma with a response rate of 40% while having little clinical effect in synovial sarcoma and myxoid/round cell liposarcoma due to many factors, including PD-L1 expression, tumour mutation burden, T-cell infiltration, etc. CTLA-4 is a key protein during the priming phase of the immune system, and its inhibition is thought to increase T cells in circulation and deplete Tregs. A blockade of the PD-1/PD-L1 axis results in a profound tumour response that could occur as early as 8–12 weeks. Thus, the combination of an anti-PD-1 and anti-CTLA-4 strategy indicates longer-lasting activity. Currently, the combination of two checkpoint inhibitors has already resulted in improved outcomes with melanoma [26]. Given its mechanism and clinical benefit in melanoma, trials involving dual blockade with PD-1/PD-L1 and cytotoxic T-lymphocyte-associated protein 4 inhibitors in sarcoma are ongoing (NCT02428192, NCT02982486). Talimogene laherparepvec (T-VEC) combined with pembrolizumab demonstrated acceptable safety and promising anti-tumor activity across a range of sarcoma histologies with 4 patients maintain PR (NCT03069378). In addition to immune checkpoint inhibitors, dendritic cell (DC)-based therapies have yet to show clinical efficacy in bony or soft-tissue sarcomas [27, 28]. An open label, phase I/II study evaluated the PFS of patients receiving autologous dendritic cell vaccine loaded with allogeneic tumor lysate expression of cancer-testis antigens in patients with STS (NCT01883518). Adoptive cellular therapy targeting NY-ESO-1-expressing synovial and myxoid/round cell liposarcoma has shown some efficacy [29, 30]. Thus, future clinical studies related to immunotherapy will hopefully further need to identify the appropriate population that will benefit from this therapy.

Importantly, novel combinations of immune-target therapies are emerging to explore whether the targeted drugs may assist in inducing consistent and durable immune responses. For example, blockade of mTOR combined with nivolumab is being tested for antitumour activity in sarcoma (NCT03190174). Axitinib, an RTK inhibitor reported to induce T-cell trafficking, is applied with pembrolizumab in sarcoma (NCT02636725). Sunitinib plus nivolumab induced objective responses in several sarcoma types with 10 patients achieved disease control (71.4%), including clear cell sarcoma (2 PR), angiosarcoma (1 PR), differentiated chondrosarcoma (1 PR), synovial sarcoma (1 PR), alveolar soft part sarcoma (1 PR) (NCT03277924).

Although our study is unique, there are some limitations in this analysis that are worth noting. First, phase I studies were excluded because of the heterogeneity of phase I trials and their typical focus on the use of safety end points. Second, ClinicalTrials.gov does not include all trials performed worldwide. Clinical trials not registered in clinical trial.gov could be found in Pubmed or through conference meetings. However, many of these trials are pilot studies and the impact is small. Additionally, trials may not have been included in our database if essential keywords were not included upon registration, and misclassification may lead to an inappropriate conclusion. However, our screening process attempted to minimize the potential biases. All the studies were reviewed by 2 physicians independently.

## Conclusions

To our knowledge, this is the first analysis to comprehensively define the landscape of metastatic sarcoma trials over the last 2 decades. A concerted effort among the NIH, industry and academia is required to increase the quality and quantity of clinical trials of sarcoma with a significant goal of improving the identification and delivery of effective therapies for patients with sarcoma.

## Additional file

Additional file 1: Information of 277 eligible Trials with ClinicalTrials.gov identification numbers in this study. (XLSX 96 kb)

## Abbreviations

CDK: cyclin-dependent kinases
CTLA-4: cytotoxic T-lymphocyte-associated protein 4
EMA: European Medicines Agency
FDA: Food and Drug Administration
GIST: gastrointestinal stromal tumours
HDAC: histone deacetylase
IGF1R: insulin-like growth factor 1 receptor
LAG-3: Lymphocyte-activation protein 3
NIH: National Institutes of Health
PD1: programmed cell death protein1
PDGFR: platelet-derived growth factor receptor
PD-L1: programmed death-ligand 1
RTKs: receptor tyrosine kinase
STS: soft tissue sarcoma
Tim-3: T cell immunoglobulin mucin-3
VEGFR: vascular endothelial growth factor receptor
WHO: World Health Organization
